# Components, prospects and challenges of personalized prevention

**DOI:** 10.3389/fpubh.2023.1075076

**Published:** 2023-02-16

**Authors:** Stefanie Jaskulski, Cosima Nuszbaum, Karin B. Michels

**Affiliations:** ^1^Institute for Prevention and Cancer Epidemiology, Faculty of Medicine and Medical Center, University of Freiburg, Freiburg, Germany; ^2^Competence Network Preventive Medicine Baden-Württemberg, Competence Area of Personalized Prevention, Freiburg, Germany; ^3^Department of Epidemiology, Fielding School of Public Health, University of California, Los Angeles, Los Angeles, CA, United States

**Keywords:** personalized prevention, precision health, nutrigenetics, nutrigenomics, predictive biomarkers, precision health policies, genetic discrimination

## Abstract

Effective preventive strategies are urgently needed to address the rising burden of non-communicable diseases such as cardiovascular disease and cancer. To date, most prevention efforts to reduce disease incidence have primarily targeted populations using “one size fits all” public health recommendations and strategies. However, the risk for complex heterogeneous diseases is based on a multitude of clinical, genetic, and environmental factors, which translate into individual sets of component causes for every person. Recent advances in genetics and multi-omics enable the use of new technologies to stratify disease risks at an individual level fostering personalized prevention. In this article, we review the main components of personalized prevention, provide examples, and discuss both emerging opportunities and remaining challenges for its implementation. We encourage physicians, health policy makers, and public health professionals to consider and apply the key elements and examples of personalized prevention laid out in this article while overcoming challenges and potential barriers to their implementation.

## Introduction

The global burden of complex heterogeneous diseases with multiple etiologies, such as cancer, diabetes mellitus type 2 (DM2), and cardiovascular diseases (CVD), is rising globally. At the same time, adverse lifestyle changes at a population level also add to risk. These trends represent a serious public health concern, and indicate a need for more effective prevention strategies. To date, most preventive strategies to reduce disease burden have targeted the general public by providing “one-size-fits-all” recommendations (1). These include most “5-a-day” to improve fruit and vegetable intake or advice to “maintain a normal body weight.” While curative medicine is increasingly embracing an individualized approach (e.g., tumor boards in oncology), preventive strategies are lagging behind. Disease risk of complex heterogeneous diseases is based on multiple genetic, genotypic, phenotypic, clinical, and environmental factors, and complex gene-environment-interactions. Recent advances in “omics” technologies have offered new opportunities to stratify individual characteristics and assess potential disease risks in more detail based on individual biological data (e.g., genetic, genomic, microbiome) (2) along with sociodemographic, clinical, and environmental factors. These technologies are likely to allow the further personalization and precision of prevention strategies. As a result of the limited success of population-based prevention strategies and the development of rapidly evolving digital technologies (3), the interest of health policy-makers and professionals in more personalized prevention strategies continues to grow. In this review, we summarize the current components that have been realized, and then we review the literature and make suggestions of additional new components how to further personalize prevention, and discuss both its potential and challenges associated with its implementation.

## Status quo of prevention

Around the world, various preventive interventions are recommended and offered as part of regular health care. In the USA, for example, individuals with health insurance are eligible for *annual physical check-ups*, which include a standardized battery of examinations and tests including weight, blood pressure, body temperature, vaccinations, screening tests, and disease (risk) biomarkers (4). In Germany, *health check-ups* are covered by public health insurance once for those under 35 years of age and every 3 years for those over 35 years of age (5). Both of these include a review of medical and family history, anthropometric measurements [i.e., body mass index (BMI)], some biomarkers if indicated (blood tests or, in the case of a conspicuous family history, genetic testing) and counseling with health advice on risk factors and lifestyle recommendations (e.g., weight reduction, dietary changes).

Screening programs are usually based on age and gender, e.g., biennial breast cancer screening by mammography for women aged 50–74 years (6) and annual screening for colorectal cancer by colonoscopy from age 45 in the USA (7) and from age 50 in Germany in 10 year intervals (8). In addition to these previously implemented approaches, a variety of other possibilities for future personalization will be reviewed in the following chapter.

## Key components of personalized prevention

**Figure 1:**
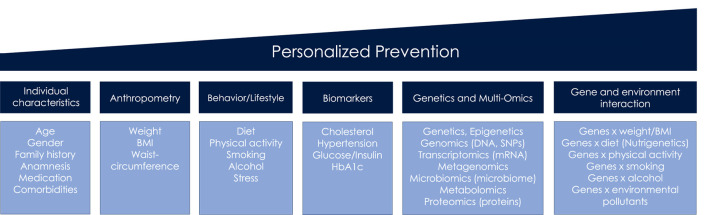
Key components of personalized prevention.

The term *personalized prevention* is used throughout the review, irrespective of alternative terminologies that have accrued including precision, individualized or targeted prevention (2). We define *personalized prevention* as approach that incorporates information on sociodemographic, clinical, anthropometrical and behavioral factors, biomarkers, omics, and gene-environmental interactions (Figure 1), in order to indicate an individual's risk for diseases. All of this information is subsequently used to personalize preventive care.

This chapter provides an overview of these key components as understood to date, and presents exemplary fields of application for personalized prevention of cancer and cardiometabolic diseases (CMD).

## Behavioral interventions

Personalized preventive behavioral interventions may be more effective than general preventive recommendations in achieving long term behavior change that might affect risk of complex diseases like DM2 and CVD (9). In this section, studies that have reviewed the effectiveness of personalized preventive counseling based on personal lifestyle data from disease prevention questionnaires are discussed and evaluated. As focus has increasingly been placed on web-based approaches, the following section will mainly deal with these techniques.

### Intervention studies on the effectiveness of personalized lifestyle-based advice

Table 1 shows randomized-controlled trials (RCTs), evaluating the effect of personalized lifestyle-based prevention advice. Personalized advice was based on dietary habits (10, 13, 16) and physical activity (10, 16), CVD (11, 12, 15) or CMD risk profiles (14), behavior (i.e., diet), and clinical changes (i.e., weight reduction, cholesterol), compared with general guidelines.

**Table 1:** Effects of personalized prevention based on current diet, physical activity or risk assessment on behavior, anthropometrics and biomarkers – An overview of RCTs.

**First author (Country)**	***n* analysis (recruitm)**	**FU RCT**	**Disease prevention**	**Inclusion criteria**	**Intervention group (IG) (*n* analysis), conditions**	**Control group (CG) (*n* analysis), conditions**	**Primary outcomes; secondary outcomes**	Main results^*^(changes with respect to behavior, anthropometrics, and biomarkers)
Gill et al. (10) (USA)	158 (288)	3 mo 6 mo 12 mo	CVD	18-65y; low a1 HDL (men <20 mg/dL; women <30 mg/dL) or high WC (men ≥ 101.6 cm, women ≥ 88.9 cm; access to the internet	Personalized diet + exercise plan *via* an online portal with online tools + a phone consultation to discuss the plan (6 mo n 81, 12 mo n 66)	Controls (NA) (6 mo n 72, 12 mo n 70)	A1 HDL; weight, body fat%, WC, glycemic control, insulin resistance, lipids, inflammation markers	♦ 3, 6, 12 mo: sig reductions in weight loss (BMI 12 mo ns) IG vs CG ♦ Sig reductions in WC (sig only 12 mo), in body fat (12 mo p=0.05) • At 3, 6, 12 mo: sig increase in a1-HDL in the IG vs VG; sig increase in a2-HDL at 3 + 6 mo, but not at 12 mo • Sig reduction in fasting insulin at 3 and 12 mo IG vs CG • Sig correlation at 6 and 12 mo betw high participation and higher a1-HDL and decrease in a4-HDL
Groeneveld et al. (11) (Netherlands)	517 (m)	12 mo	CVD	18-65 y; male industry workers; elevated risk of CVD; > moderate 10-year CHD risk; ≥ 1 additional risk factor	Face-to-face + telephone counseling on CVD risk, discussing information, behavior change + goal setting (*n* 261)	Brief verbal & written information about their CVD risk profile (n 256)	Body weight, BMI, systolic, diastolic BP, HDL chol, total chol/HDL chol ratio, HbA1c	♦ Sig weight loss • Sig effect on HbA1c value and systolic blood pressure • Sig increase in HDL cholesterol • Sig reduction in cholesterol ratio
Groeneveld et al. (12) (Netherlands)	595 (m)	12 mo	CVD	18–65 y; male industry workers; elevated risk of CVD; > moderate 10-year CHD risk; ≥ 1 additional risk factor	Face-to-face + telephone counseling on CVD risk profile; discussion of a desired topic (PA, diet or smoking); motivation, goal setting; supervision (*n* 288)	Usual care and information about CVD, PA, diet and smoking (*n* 307)	PA (SQUASH), diet (Short Questionnaire for Measuring Fruit and Vegetable Intake), smoking	■ Ns effects on PA ■ Sig improvement in snack consumption ■ fruits and vegetables ns difference ■ Sig reduction in alcohol consumption among normal-weight ♢ Sig improvement on smoking
Rijnaarts et al. (13) (Netherlands)	79	3 mo	healthy adults	≥18 y; healthy; low fiber intake [w <26 g, m <33 g (FFQ)], living in the Wageningen area, mobile phone with applications	Personalized dietary advice through information on a website, in addition to general nutrition recommendations (*n* 34)	General dietary advice by means of information flyer; after 6 wks: also access to the website (*n* 45)	Dietary fiber intake (247-item semi-quantitative meal-based online FFQ)	■ Sig more participants in the IG met the recommended dietary fiber intake after 6 wk vs. CG; sig lower fiber intake on weekends of wk 1 and wk 6 than during the week (for both groups) ■ Sig higher fiber intake at 3 mo in both groups. ♦ Ns change in body weight ♢ IG rated counseling and advice as sig more positive
Stol et al. (14) (Netherlands)	1934; 63 y (Mean)	12 mo	CMD	45–70 y; no CMD, a CMD risk factor, or anti hypertensive, lipid lowering or antidiabetic treatment (health record)	CMD prevention program, risk score, BP measurement, laboratory testing, persona-lized lifestyle counseling + treatment (*n* 967)	Completion of a health questionnaire; after 1 year: invitation to the CMD preven-tion program (*n* 967)	Newly detected CMD or started drug treatment, mean change in individual CMD risk factors, change in absolute 10y CVD mortality risk (SCORE-EU)	♦ Sig reduction in WC • Sig reduction in systolic BP, total cholesterol, cholesterol ratio, and LDL in the IG vs CG • Ns differences for changes in BMI, diastolic BP, fasting glucose, and smoking status
Khanji et al. (15) (United Kingdom)	377	6 mo.	CVD	40–74 y, 10-y QRISK2 CVD risk score ≥10%, access to the internet, email, mastery of the English language	Web-based e-coaching with personalized lifestyle + risk score, information + advice, regular reminders of goals by mail (*n* 194)	Personal counseling on lifestyle, risk + protective factors + personal lifestyle questionnaire (*n* 183)	Pulse wave velocity change; PA (RPAQ), BP, weight, chol, glucose, hsCRP, quality of life, 10-y Framingham Risk score, etc.	■ Improvement in alcohol intake in both groups (ns differences) ■ Improvement in PA in both groups (ns differences) ♢ Ns difference in pulse wave velocity reduction • Improvements in BP and cholesterol levels in both groups (ns) ♦ Improvements in BMI and WC in both groups (ns)
Anderson et al. (16) (Scotland)	59: (9 m/69 w)	12 wk	Weight management	≥18 y, family history of colorectal or breast cancer, ≥25 kg/m^2^	Individualized diet, persona-lized activity, behavioral change techniques + consultations (*n* 30)	Usual care, lifestyle booklet (*n* 29)	Feasibility measures; measured changes in weight + PA, reported diet, psychosocial measures	■ Sig lower fat consumption score betw IG vs CG ■ Unsaturated fat and fiber food consumption score ns ■ Sig higher daily average time spent in moderate PA IG vs CG ♦ Sig lower body weight, BMI and WC IG vs CG • Mean systolic and diastolic BP sig lower in IG, but ns vs CG

* Focus on results regarding the mean difference between intervention group(s) (IG) and control group (CG) (mean difference to baseline) - statistically significant (sig) difference if p <0.05; not significant (ns p≥0.05).

BMI, body mass index; BP, blood pressure; CG, control group; CMD, Cardiometabolic disease; CVD, cardiovascular diseases; CHD, coronary heart disease; chol, cholesterol; diff, differences; FU, follow up; HDL, high-density lipoprotein cholesterol; LDL, low-density lipoprotein cholesterol; IG, intervention group; m, man; mo, months; n, number of participants; ns, not significant; WC, waist circumference, PA, physical activity; RCT, randomized controlled trial; sig, significant; w, women; wk, week(s); y, years.

■ Behavioral changes in diet; □ Behavioral changes in physical activity; ♦ Changes in body weight/BMI/WC; • Changes in biomarker concentrations; ♢ Other changes.

With respect to dietary changes, a personalized 6-month lifestyle intervention for moderate CVD risk resulted in significant reductions in snack and fruit intake (12). Another RCT indicated that a patient group receiving personalized dietary advice on fiber intake exhibited higher adherence than a control group receiving general advice (13). A meta-analysis including 18 RCTs (1998–2011) from the USA, Netherlands and Belgium revealed higher effectiveness of e-Health personalized lifestyle interventions in increasing fruit and vegetable intake when compared to general recommendations (17).

A 12-month-intervention found significant improvement in smoking reduction only at 6 months with no effects of personalized prevention observed for physical activity (12). Furthermore, a significant reduction in alcohol consumption compared to control groups was seen only among normal-weight participants (12). In two prospective trials from the Netherlands, personalized e-Coaching lifestyle interventions led to a 20–25% relative reduction in 10-year CVD risk. However, a RCT did not reveal any effect (18).

In some studies personalized lifestyle-based interventions led to significantly greater reductions in body weight (10, 11, 16), and waist circumference (WC) (10, 14, 16), compared to the controls. However, this was not observed in other studies (13, 15). An older meta-analysis of 12 RCTs (2001–2012) targeting web-based diet and/or physical activity interventions for weight loss suggested a greater reduction in weight, BMI and WC in participants receiving web-based delivered personalized feedback compared to controls. Significant differences were apparent at both 3–6 months. These results were not found in studies with durations of 12–24 months (19).

In two studies the interventions had a significant effect on increasing high-density lipoprotein (HDL) cholesterol (10, 11). A significant reduction effect in systolic blood pressure (BP) between the groups was also observed in two studies (11, 14), but not in others (15, 16). The intervention in two RCTs led to significant reductions compared to controls in triglyceride and insulin concentrations (10) and in hemoglobin A1c levels (HbA1c) (11), a marker indicating prediabetes.

Two of the aforementioned studies (14, 15) used a stepwise prevention program for CMD that was comprised of initial individual risk assessment *via* validated disease risk score using a comprehensive online questionnaire (step 1), followed by additional risk profiling through laboratory tests, visiting general practitioners in case of high-risk individuals (step 2), and providing web-based individualized lifestyle advice (step 3) (10, 14). A large RCT with 967 participants with increased CMD risk and 967 controls demonstrated significant long-term decrease in WC, systolic BP, cholesterol, and LDL in the intervention compared to the control group (14). Results from the other RCT did not provide evidence of beneficial effects following personalized lifestyle counseling compared to standard of care (Table 1) (15).

### Evaluation

The findings suggest that the provision of lifestyle-based risk advice might be a more useful tool for behavioral lifestyle changes compared to the use of general recommendations (1, 10–14, 16–19). However, it is not clear whether this increased motivation can be sustained over the long term (1, 11, 12, 19). Beyond that, the detection of high-risk individuals identified by disease risk scores based on sociodemographic, clinical, anthropometrical and behavioral factors, might be an effective strategy for those individuals who could most benefit from prevention strategies (14).

## Biomarkers as intervention targets

Preventive approaches targeting clinical biomarker measurements are also an important pillar of personalization that already have been partly realized. Cardiometabolic biomarkers (including blood lipids, glucose, HbA1c) from laboratory testing in several combinations may provide a primary risk profile and indicate preliminary stages of CMD and their phenotypes, particularly when additional information like anthropometrics, sociodemographics, and/or familial history (Figure 1) is considered (3, 20, 21).

Improving clinical biomarkers can be achieved by various means including weight loss, physical activity, and nutritional adjustments. Nowadays, a steady expansion of the product range and marketing of nutritional supplements means that more and more consumers are taking supplements, regardless of whether or not they actually exhibit a deficient or insufficient supply of minerals, vitamins and micronutrients. A targeted supplementation of food supplements tailored to the individual deficiency should occur only when based on laboratory diagnostics.

Moreover, there is increasing evidence that the human gut microbiome plays a crucial role in immune function, human health, and disease. There is evidence to suggest that stratification of individuals according to two microbial enterotypes (either Prevotella or Bacteroides dominance) may be valuable in predicting responses to diets for obesity management (22). Furthermore, the influence of the gut microbiota on human health may be in part mediated by the ability to metabolize dietary compounds into new bacterial metabolites that may impact disease risk (23). For example, short chain-fatty acids (SCFA) produced by gut bacteria may provide a mediating link between dietary fiber intake and metabolic diseases (24).

Personalized nutrition guidance that involves biomarkers identified by various omics technologies, self-reported questionnaire-based dietary assessment tools, and useful tools to improve real-time assessment of dietary intake and feedback provision (e.g., by wearable devices and mobile apps), may have the potential for more effective glycemic control and prevention of DM2. A machine-learning algorithm that integrates particular dietary habits (using a smartphone-adjusted website), blood parameters, gut microbiota and continuous glucose monitoring was effective to predict postprandial glycemic responses to real-life meals more accurately than general dietary advice (25).

In addition, metabotyping is a new concept in which individuals are clustered into metabolic phenotypes based on clinical and biochemical parameters, anthropometric measures, metabolomics and metagenomics data. This concept, which aims to tailor diets that fit each metabotype specifically, has been suggested as a nutritional strategy, for example in the context of CMD prevention. However, health economics studies are still lacking (23).

### Evaluation

As part of ongoing technological progress including molecular diagnostics, more and more biomarkers are likely to be discovered. The combination of biomarkers with sociodemographic, anthropometric, clinical and behavioral factors, and omics data can help with the identification of individuals at high risk and the detection of even pre-symptomatic chronic diseases.

## Genetics, genomics and epigenetics

Interest in identifying individual genetic biomarkers as a strategy to help achieve a high degree of personalization in prevention is currently increasing. One approach is the analysis of monogenetic genes to identify increased risks for developing specific diseases. An examination of genes for coding variations likely to make individuals susceptible to breast cancer revealed an association between breast cancer risk and protein-shortening variants of these genes in ATM, BRCA1, BRCA2, Check2, and PALB2 (26).

Genome-wide association studies (GWAS) assess genetic variants across the genomes of a large number of individuals to identify genotype–phenotype associations using the technologies of whole genome sequencing and the determination of single-nucleotide polymorphisms (SNPs). Furthermore, GWAS enable the calculation of polygenic risk scores (PRS) as a measurement of risk summed over multiple risk alleles (27). In one study, a PRS for the prediction of estrogen receptor-characteristic breast cancer was developed based on 69 studies and a large data set of GWAS. Here, the optimal PRS was able to significantly predict the risk of developing breast cancer and consisted of 313 SNPs (28).

GWAS studies investigating genetic variants associated with obesity and weight loss resistance have identified several potentially relevant variants. These include the obesity predisposing FTO (fat mass and obesity associated gene) (29) and MC4R (melanocortin 4 receptor) genes related to food intake control (e.g., appetite, eating behavior) and energy homeostasis regulation (30).

However, a limitation of GWAS is the missing impact of the environment and identifying gene-environment interactions, which are both assumed to have a high influence on the individual disease risk (27, 31).

The DNA cannot be changed directly, but the function of individual genes can be influenced by epigenetic mechanisms. Epigenetics is one section of genetics which describes the variations in gene expression without a change in the DNA sequence itself (32). One main carrier of epigenetic information is DNA-methylation (33). Epigenetic alteration can cause pathologies, including cancer (31). Epigenome-wide association studies (EWAS) focus on the influence of environmental factors on the underlying mechanisms related to the development and progression of diseases (34).

### Evaluation

Genetic and genomic testing seems promising for people having a family history of genetic conditions. The implementation of epigenetics has an even bigger potential for personalization due to its modifiability and the possibility of adaptations through environmental interactions.

## Gene-environment interaction

Gene and environment interactions (GxE) determine the responses of individuals to environmental variation based on their (epi)genetic profile (31). Those variations can include diet and lifestyle factors.

Obesity represents a major risk factor for CMD like CVD and DM2, all of which exhibit complex interplay mechanisms of genetic and environmental risk factors (30). GxE studies have revealed evidence for decreased risk of some genetic predispositions (for example toward obesity) through the interaction between protective nutritional factors and higher levels of physical activity (30, 35, 36).

Nutrigenetics studies how genes affect nutrient metabolism (37). Large prospective studies found that increased intake of both sugar-sweetened beverages and fried food amplified the association of an obesity-related 32-SNP genetic risk score (GRS) with BMI (35). Dietary fat intake modified the association between the FTO genotype and changes in insulin sensitivity and obesity. FTO carriers exhibited a greater reduction in weight and fat distribution in response to a high-protein diet (36). While some studies observed significant interactions influencing BMI between the FTO variant and higher dietary intake of calories, proteins, saturated fat, carbohydrates and salt, one large study with 177,300 adults did not find significant interactions (35). The results of a case-control study including 7,052 high cardiovascular risk subjects suggested that a high adherence to the Mediterranean Diet counteracts the genetic predisposition toward DM2 related to FTO and MC4R (36). Furthermore, a meta-analysis revealed that higher levels of physical activity had a significantly attenuating effect on an obesity-associated 12-SNPs GRS in the American but not in the European cohorts (35).

### Intervention studies on the effectiveness of phenotype- or genotype-based lifestyle advice

Table 2 summarizes key RCTs, published between 2013 and 2021, that assess the effectiveness of the provision of phenotype or genotype-based lifestyle advice on changes in anthropometrics, behavior, clinical results, and psychological factors.

**Table 2:** Effects of personalized prevention based on current diet, phenotype and/or genotype on behavior, anthropometrics and biomarkers – An overview of RCTs.

**First author (Country)**	***n* analysis (*n* recruitm): *n* m/w; mean age; BMI ±SD^†^**	**FU**	**Setting for personalized prevention**	**Intervention group(s) (IG) conditions (*n* analysis)**	**Control group (CG) conditions (*n* analysis)**	**Primary outcome(s); secondary outcome(s)**	Main results^*^changes with respect to behavior, anthropometrics, biomarkers, psychological factors (e.g., motivation, anxiety)
Celis-Morales et al. (1) (Ireland, Spain, Poland, Netherlands, UK, Greece, Germany)	1269 (1488): 618 m/870 w); 39.8 y (18 – 79); 25.5 kg/m^2^ ± 4.9	3 mo, 6 mo	Genotype-based advice related to metabolism	- L1: Dietary advice based on current diet (specific food groups, reported nutrients intake) (*n* 312) - L2: L1 + phenotype (*n* 324) (BMI, WC, blood nutrient-/metabolic biomarkers (omega-3 index, carotenoids, glucose, cholesterol)) - L3: L2 + genotype (*n* 321) (genotype-based information - 5 genes: *FTO, ApoE (e4), FADS1, MTHFR, TCF7L2*)	L0: non-personalized dietary advice based on conventional population-based healthy eating guidelines (*n* 312)	Dietary intake (online-FFQ, Healthy Eating Index (HEI)); Body weight, BMI, WC	Effect on intakes of major food groups: IGs (L1, L2, L3) vs. L0 (sig p <0.05): mo 3 and 6: ■ Sig less red meat, less salt, lower energy intake, higher HEI scores; changes in dietary outcomes did not differ betw L1, L2 and L3 of PN Effect on dietary outcomes: IGs (L1, L2, L3) vs. L0 ■ Mo 3: sig improvements for salt, saturated fat, blood carotenoids ■ Mo 6: sig less salt and saturated fat, higher folate intake ■ Changes in dietary outcomes did not differ betw L1, L2, and L3 of PN Effect of targeted intervention on anthropometric outcomes: IGs (L1, L2, L3) vs. L0: ♦ Mo 3: sig improvements for body weight and BMI; ns at 6 mo
Livingstone et al. (38)°	1270 (1480): 59% female; 39.9 y ± 13·0; BMI 25.5 ± 4.9	3 mo, 6 mo	Genotype-based advice related to metabolism	- L1: Personalized nutritional counseling based on current diet (*n* 312) - L2: L1 + phenotype (*n* 325) - L3: L2 + genotype (*n* 321)	L0: Dietary recommendation based on general guidelines (*n* 312)	Dietary intake (online-FFQ)	At 6 mo: ■ MedDiet scores sig greater in individuals with PN (L1, L2, and L3) than in CG ■ Ns difference in MedDiet scores betw. L1 vs. L2 +L3 ■ Differences in MedDiet scores sig greater in L3 than in L2 ■ Effect of PN intervention was sig better for Med countries (Greece, Spain) than for the non-Med countries
Livingstone et al. (39)°	1270 (1480): 59% female; 40.9 y ± 13.0; 25.4 ± 4.8	3 mo, 6 mo	- Genotype-based advice related to metabolism	- L1: PN counseling based on current diet (*n* 312) - L2: L1 + phenotype (*n* 325) - L3: L2 + genotype (*n* 321)	L0: Dietary recommendation based on general guidelines (*n* 312)	Dietary intake (online-FFQ)	Between PN (L1+L2+L3) (n=958) vs. L0 at mo 6: ■ Discretionary intake, FSS (Food Standards Scotland): ns differences from discretionary food items (except total sugars p <0·05) ■ ADG (Australian Dietary Guidelines): sig greater reductions in energy, total fat, saturated fat and total sugars in PN groups vs. L0 ■ % contribution to intake of total sugars from sweets and snacks lower in PN vs. L0
Marsaux et al. (40) (Ireland, Spain, Poland, Netherlands, UK, Greece, Germany)	1233 self-reported data on PA (730 objective PA/ACC) (1480): 615 m / 865 w; 39.9 y ±13.0; NA	3 mo, 6 mo	Genotype-based advice related to metabolism	- L1: personalized advice based on individual dietary intake + PA (self-reported PA 304/ objective PA 176) - L2: L1 + phenotype (self-reported PA 309/ objective PA 188) - L3: L2 + genotype (self-reported PA 315/objective PA 196)	L0: general advice based on guidelines for diet and PA (self-reported PA 305/objective PA 170)	Physical activity (ACC, PA questionnaire); dietary intake (online-FFQ)	*Self-reported PA:* □ 6 Mo: sig greater improvement in self-reported total PA and PA during leisure (nonsport) in personalized groups (L1-L3) vs L0 □ For individuals advised to increase PA: sig improvements in all IGs; sig greater improve- ments in 2 self-reported indices with increased personalization of advice (L2 + L3 vs L1); L3 sig higher moderate exercise than L2 *Objective PA (*accelerometer)*:* □ In ACC results: ns differences betw L1-L3 vs. L0 and ns effect of adding phenotypic or genotypic information to the tailored feedback at mo 3 or 6 □ 6 mo: small but sig improvements in the objectively measured PA level, moderate PA and sedentary time for individuals advised to increase PA (similar in all groups) □ 6 mo: sig reduction in sedentary activities for all groups (ns betw groups)
Nielsen and El-Sohemy (41) (Canada)	123 (138): 32 m/ 106 w; 26.5 y ±3.0; NA	3 mo 12 mo	Genotype-based advice related to metabolism; healthy	Personalized dietary advice (for caffeine, vitamin C, added sugars + sodium) based on genotype *(CYP1A2, GSTM2 +* GSTT1, TAS1R2, ACE) (*n* 82)	General dietary advice (*n* 41)	Dietary intake (FFQ); meeting recommendations	■ 3 Mo: ns effects in the ingested amount of caffeine, vitamin C, sugar, and sodium in the IG vs CG ■ 12 Mo: sig reduction in sodium intake in the IG compared to the CG
Roke et al. (42) (Canada)	56 (57): 57 w; 22 y ±1.5; NA	12 wk	Genotype-based advice related to metabolism	Genotype *(FADS1)-*based advice (n 28)	General advice	Dietary intake (FFQs), biomarkers, anthropometrics	■ Ns differences in dietary intake of omega-3- fatty acids between IG and CG (sig increase in intake of EPA and DHA in both groups) ■ More knowledge about omega-3 terminology + abbreviation in the IG compared to the CG • Ns differences in triglycerides, Chol, HDL, Chol/HDL ratio, LDL, Non-HDL Chol.
Horne et al. (43) (Canada)	112 (140): 18 m/ 122 w; 53.5 y ±13.6; IG 37.3 ± 9.7, CG 36.7 ± 7.3	3 mo 6 mo 12 mo	Genotype-based advice related to metabolism; BMI ≥25 kg/m^2^	Diet advice based on genotyping *(UCP1, FTO, TCF7L2, APOA2, PPARγ 2, MC4R);* nutrigenomics-guided lifestyle intervention programme (*n* 59)	Nutrition plan with controlled calories and less low-fat (25%) (*n* 53)	Change in dietary intake (3 × 24 h recalls, multiple-pass-method)	12 mo: ■ Sig long-term reduction in total fat intake in the IG vs CG ■ Sig reduction of unsaturated fatty acids in grams in the IG in contrast to the CG ■ Dietary adherence to total fat + saturated fat guidelines sig greater IG vs. CG ■ Sig greater adherence to protein intake in the CG vs IG
Meisel et al. (44) (UK)	279 (1016): 138 m/141 w; IG 20.2y ±2.5, CG 20.9 ±3·0); IG 21.2 ±2.5, CG 21.4 ± 2.6	1 mo	Genotype-based advice related to metabolism	Weight control advice and genetic test feedback *(FTO gene)* (*n* 139)	Weight control advice only (*n* 140) (at later date: genetic feedback)	Readiness to control weight (validated measure of readiness for behavior change); frequency of adherence to each tip	° Sig higher motivation (consideration or action phase of weight loss) in the IG vs. CG ° Sig higher willingness to control weight in the IG vs CG ° High risk subjects in the IG were more likely to be in the contemplation phase than those at low risk; ns difference between low risk subjects and CG. ♦ Sig interaction between weight + group; in the IG, overweight/obese subjects more often in the consideration and action phase than the normal-weight subjects in the same group
Godino et al. (45) (England)	550 (569): 268 m/301 w; 48.7 y ±7·3; BMI 26·1 ± 4·2	8 wk	Genotype-based advice related to DM2 risk	Lifestyle advice with - A phenotypic-based T2D risk estimate (Cambridge Diabetes Risk Score) (*n* 182) or - Genotype-based T2D risk estimate *(23 diabetes SNPs)* (*n* 184)	Lifestyle advice with no risk estimate (*n* 184)	PA energy expenditure (kJ/kg/d) (heart rate monitor + ACC); self-reported diet (12 fruits items & 26 vegetables, FFQ), self-reported weight, worry, anxiety, perceived risk to get T2D in the next 10 years	■ Overall, ns betw-group differences on diet (genetic risk group vs. CG; phenotypic risk group vs. CG, genetic vs. phenotypic risk group). □ Overall, ns between-group differences on PA □ Genetic risk group vs. CG: greater increase in PA among women than men; phenotypic risk estimate vs. CG. no differential effect by sex ♢ Estimates of perceived risk sig more accurate among those who received risk information (genetic, phenotypic risk estimate) vs. CG ♦ Ns differences in self-reported weight effects ° Ns differences in worry at FU, and in behavioral intention and anxiety postintervention or at FU between trial groups
Voils et al. (46) (UK)	601 (3621): 483 m/118 w; 54·1 y ±8·7; NA	3 mo 6 mo	Genotype-based advice related to DM2 risk; BMI ≥27 kg/m2; veteran outpatients	Diabetes risk estimates + gene test result (CR+G) (DM2-related genes *TCF7L2, PPARγ, KCNJ11*) (*n* 298)	Non-DNA-Diabetes risk estimates plus control eye disease counseling (CR+EYE) (*n* 303)	Weight 3 mo; weight 6 mo, daily PA (self-reported PA > moderate PA, walking), dietary intake (2000 FFQ), insulin resistance, perceived risk	□ No differences in PA at 3 or 6 mo ■ At 3 mo: calorie (energy, kcal) (p=0·05) and monounsaturated and polyunsaturated fat intake (both p <0·05) sig lower in the CR+G arm vs CG, but not at 6 mo • Ns carbohydrate, protein, total fat and saturated fat intake ■ Ns differences in weight • Ns differences in insulin resistance ♢ Ns differences in perceived risk at 3 or 6 mo
Hietaranta-Luoma et al. (47) (Finland)	107(296): 33 m/ 74 w); 47·0 y ±12·1; NA	10 wk 6 mo 12 mo	Genotype-based advice related to CVD risk; healthy individuals; BMI 20 - 35 kg/m^2^	ApoE genotype risk for CVD (*ApoE*) - A high-risk (ε4+): Emphasis on dietary changes + exercise for genotype (*n* 16) - A low-risk (ε4–): Emphasis on interaction of genotype with environmental + lifestyle factors (*n* 35)	General Information about health, lifestyle and nutrition recommendations (*n* 56)	Behavioral changes in diet (e.g., fat quality, vegetables, berries, fruits, fatty & sugary foods), alcohol, leisure time PA (self-reported), Health and Taste Attitude Scales	■ Sig higher intake of unsaturated fatty acids and reduced intake of saturated fatty acids in the ε4+ group vs. CG ■ Sig improvement in ε4+ and ε4- in dietary fat quality betw FU time points vs. T0 and betw ε4+ and the CG; no interaction effect (time x group) ■ In the ε4– group, the consumption of fatty and sugary foods sig decreased the most over the time points vs CG (sig. interaction effect time x group) ■ All groups ate more fruits, berries and vegetables portions/day; ns group effects □ Ns intervention effects on leisure time PA
Sparks et al. (48) (USA)	213 (238): 56 m/182 w; 28-70 y; PRE-RA 27·4 ± 6·0, CG 27·2 ± 6·2	6 wk 6 mo 12 mo	Genotype-based advice related to for Rheumatoid arthritis (RA) risk	PRE-RA group: - PRE-RA arm: Individualized education and tips about rheumatoid arthritis based on genotype *(HLA-DRB1)* + autoantibody (*RF/CCP)* (*n* 71). - PRE-RA arm Plus: PRA-RA plus one-on-one interview for interpretation & behavior change through motivational interviewing (*n* 70)	Standard education about rheumatoid arthritis (*n* 72)	PRE-RA group and comparison arm: ladder score (smoking, diet, exercise, or dental hygiene); PRE-RA Plus arm to the PRE-RA arm on motivation (range 0–10), long-term effect 12 mo FU	At 6 mo ■ Sig. increase in fish in PRE-RA group in total & both arms separ. (higher in plus arm) vs. CG ■ Higher consumption of fruits in both PRE-RA arms vs. CG (PRE-RA arm combined vs. CG ns) ■ Ns improvement in diet ladders in the PRE-RA group vs. CG □ PA ns; ns improvement in exercise ladders in the PRE-RA group vs. CG ° Sig increased motivation in behavior change betw PRE-RA group in total vs. CG (separated: sig for PRE-RA arm cs. CG; ns for PRE-RA Plus arm vs. CG) ° Higher motivation in behavior change in the PRE-RA arm with high lifetime RA compared to CG; ns for low lifestyle RA on PRE-RA vs. CG ♢ More PRE-RA subjects quit smoking (ns)
Grant et al. (49) (USA)	108 (116); 57 y ±10·6; 34·8	12 wk	Genetic risk score assessment for DM2 prevention, overweight individuals	Genetic testing - genetic risk score calculated from 36 genotyped risk alleles associated with DM2: - High genetic risk (*n* 42) - Low genetic risk (*n* 32) Diabetes Prevention Program plus individual genetic counseling interview, behavior modification	No disease risk estimates; Diabetes Prevention Program also for untested control subjects (*n* 34)	Motivation, behavioral changes (number of sessions attended), weight change	° Ns increased motivation with respect to weight loss, diet, exercise, diabetes prevention in the high or low genetic risk group vs. CG (ns difference, except for exercise) □ Lower-risk group had sig less intent to exercise (stage of change for exercise) vs. CG ♦ IGs lost slightly more weight (ns difference from the CG) *Secondary analysis of higher- vs. lower-risk intervention arms:* ° High risk group found genetic counseling sig more motivating to participate in the 12-wk program than the lower risk group ♢ The low risk group thought sig less about their genetic risk than the high risk group
Kullo et al. (50) (USA)	203 (216): 97 m/ 106 w; 59·4 y ±5·0; IG 30·2 ± 6·1, CG 30·5 ± 7·0	3 mo 6 mo	Genetic risk score assessment for CHD prevention	Conventional + genetic risk score (GRS) (genotyping of 28 CHD SNPs) for 10 y CHD risk (n 103) - Average/low GRS (*n* 50) - High GRS (*n* 53) 30-min CHD risk counseling session, visit physician decision statin use	Conventional risk score for the 10 y CHD risk; 30-min CHD risk counseling session, visit physician decision statin use (*n* 100)	LDL-C, dietary fat + PA (screener22, TAPA), anxiety (STAI), statin use	At 6 mo ■ Ns difference between groups in dietary fat intake □ Ns difference between groups in PA • Sig lower LDL-C levels in the IG (especially in subjects at high GRS) vs. CG ° Ns difference between groups in anxiety ♢ Statins were sig initiated more often in the GRS group vs. CG

† n analysis (=number of participants included for analysis of differences between IG and CG) (n recruitm) (=number of recruited participants): n m/w (=number of participants differentiated by men/women); mean age ± SD; BMI ± SD^†^ (=mean age of participants ± SD); mean BMI in kg/m^2^ ± SD (for whole study group, used for analysis or based on recruitments/randomizations); if no given data for whole study group, then information separated for IG and CG.

* Focus on results regarding the mean difference between intervention group(s) (IG) (receiving personalized intervention) and control group (CG) (mean difference to baseline)—statistically significant (sig) difference if p < 0.05 IG compared to CG or 95% confidence interval not including effect zero; not significant (ns p ≥ 0.05).

°Secondary analysis of the Food4Me study; details in Celis-Morales et al. (1).

ACC, accelerometer; betw, between; BP, blood pressure; BMI, body mass index; CG, control group; CHD, coronary heart disease; FFQ; FU, follow up; HDL, high-density lipoprotein cholesterol; HEI, healthy eating index; IG, intervention group; LDL, low-density lipoprotein cholesterol; MedDiet, mediterranean diet; mo, months; n, number of participants; NA, not applicable; ns, not significant; PA, physical activity; RA, rheumatoid arthritis; RCT, randomized controlled trial; recruit, recruitments; SD, standard deviation; sig, significant/significantly; STAI, state and trait anxiety inventory for adults; TAPA, telephonic assessment of PA; T2D, diabetes mellitus typ 2; UK, United Kingdom; WC, waist circumference; wk, week(s).

■ Behavioral changes in diet; □ Behavioral changes in physical activity; ♦ Changes in body weight/BMI/WC; • Changes in biomarker concentrations; ° Changes on motivation, anxiety; ♢ Other changes.

To date, the Food4me study (1) is the largest and most complex RCT, including 1,269 adults from seven European countries. This study assesses the effectiveness of three different web-based types of personalized nutrition advice on changing eating patterns (compared to generalized dietary advice). The results of the study's 6-month intervention period demonstrated that personalized advice based on the individual's dietary intake and weight was more effective in motivating participants to make healthy changes to their usual diet than the conventional dietary recommendations received by the control group. However, no evidence of enhanced effectiveness from the use of more in-depth personalization in terms of phenotypic and genotypic information was observed (1, 39). Secondary analyses pointed to significantly lower sugar intake (39) and greater Mediterranean Diet scores (MDS) (38). Interestingly, differences in MDS were significantly greater in participants receiving dietary advice based on current diet, phenotype and genotype than in participants providing advice based on current diet plus phenotype only (38).

Further RCTs exhibited significant reductions in sodium intake (41), and total fat and unsaturated fatty acids (43), in the metabolic genotype-based dietary advice group compared to controls. In another RCT, no significant differences in dietary intake of omega-3-fatty acids and biomarkers (e.g., triglyceride, cholesterol) between the groups were observed (42). Following the provision of genotype-based lifestyle advice related to disease risk, RCTs found significant improvements on fat intake (46, 47), reductions in sugar intake (47), and an increase in fruits and fish consumption (48) compared to the controls. However, another RCT observed no differences in any of the food groups resulting from personalized nutrition advice (45).

Additionally, data from the Food4me study did not find evidence for higher effectiveness of personalized web-based advice on increasing physical activity (when measured *via* accelerometer) compared to the use of general guidelines (40). In contrast, self-reported physical activity significantly increased to a greater extent with higher degrees of personalized advice (40). Further RCTs revealed no significant effects of genotype-based lifestyle advice on physical activity [based on self-reports (46–48), or accelerometer (45)] compared to the controls.

With respect to weight reduction, in the Food4me study, significant effects on weight loss made by participants receiving any type of personalized nutrition were significant at three, but not at 6 months. These changes did not differ based the type of personalization (1). In contrast, no significant effects on weight between the genotype- (45, 46) or phenotypic-based risk communication group (45) compared to the control groups were observed in other studies (45, 46).

A revised version of a Cochrane review and meta-analysis from 2016 (including 18 RCTs published until 2015) did not reveal significant effects of communicating DNA based risk estimates on diet, physical activity, smoking cessation, alcohol use, or behavioral support programs (51). A cohort study including 2,037 participants, who received genomic data with provided estimates of lifetime risk of developing 18 common health conditions, did not indicate significant changes on dietary intake or exercise following delivery of genomic profiling (52). Furthermore, surveys on direct-to-consumer genetic testing (DTC-GT) have suggested that disease-specific information on genetic predisposition may motivate individuals slightly more in terms of diet and physical activity (53).

The evidence regarding the effectiveness of personalized prevention in the long-term (≥6 months) during the intervention phase is not clear. While in some RCTs only one follow-up time point was recorded and analyzed [ ≤ 3 (42, 44, 45, 49) and 6 months (38, 39)], some studies also analyzed the effects over time with two [3 and 6 months (1, 40, 46, 50); 3 and 12 months (41)] or three analyzed time points (43, 47, 48) (Table 2). With regard to RCTs assessing changes in dietary behavior and weight, some studies pointed to significant improvements in at least one outcome both in the short- and long-term [total fat, unsaturated fatty acids (43); red meat, salt, energy intake, Healthy Eating Index, saturated fat (1)]. On the other hand, different RCTs indicated significant improvements only in the short-term but not in the long-term [blood carotenoids, body weight (1); fat intake (46)]. However, other studies let to significant improvements in the long-term but not in the short-term [folate intake (1); sodium intake (41)].

### Impact of genetic risk communication on motivation and adverse effects

The motivational effect of personalized prevention can be directly measured by lifestyle changes and improvements in biomarkers. However, few RCTs have investigated the motivational effect of genetic- or genotype-based communication interventions on personal motivation for lifestyle changes (44, 48, 49) (Table 2). The older Cochrane meta-analysis of RCTs did not conclude that communicating DNA based risk estimates had any measurable effect on motivation or intention to change behavior (51). However, more recent RCTs (44, 48) have concluded that the provision of personalized genotype-based lifestyle advice can increase the motivation of participants to integrate changed behavior into their daily life. Furthermore, studies investigating whether or not the motivational effect of personalized prevention last beyond the intervention are still lacking. However, web-based approaches including techniques and reminders might support long-term behavior changes for CMD prevention. One current systematic review concluded that telemedicine platforms may be used for regular check-ups for preventive care of CVD (54).

With respect to possible adverse effects, two RCTs have reported that communication of genetic risk does not seem to increase anxiety (45, 50). Neither the results from the Cochrane meta-analysis nor the results from an online survey following DTC-GT indicated an impact of genetic risk communication on levels of depression (51) and anxiety (51, 52).

### Evaluation

In summary, the largest study to date (1) revealed that personalized nutrition advice let to healthier diet compared with controls, despite no evidence of any additional benefit from using more in-depth personalization in terms of phenotypic and genotypic information were demonstrated. However, some smaller studies reported that the provision of genetic- or genotypic based risk advice improved dietary behavior in one or more dietary outcomes compared to general advice (38, 39, 41, 43–50). Yet, the long-term effects of web-based personalized preventive interventions are still unclear. Therefore, personal contact and guidance *via* face-to-face or web-based human-delivered counseling seem to be essential. The presented findings also suggest that the provision of a genetic-based risk advice may motivate healthy behavior changes, and that communication of genetic risk does not arise to predict adverse effects like anxiety.

## Prospects of personalized prevention

**Figure 2:**
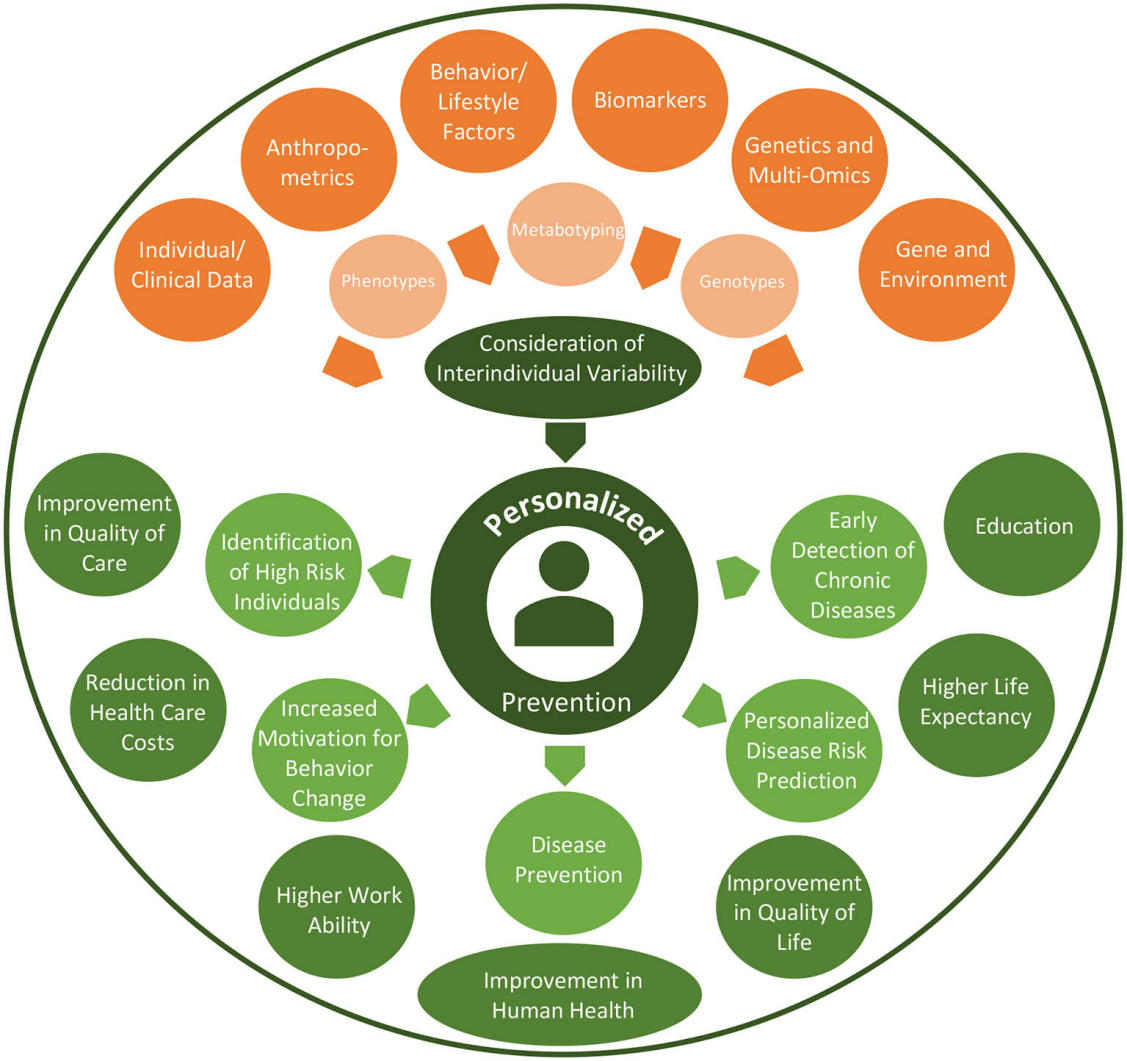
Prospects of personalized prevention.

Personalized prevention offers promising opportunities as summarized in Figure 2. Personalized prevention can help with the prediction of disease occurrence and can be implemented in ways that are targeted to an individual profile of genomic data and other biomarkers (55). In addition, personalized preventive approaches can lead to an improvement in the quality of care and quality of life of patients and furthermore to a reduction in healthcare time, effort and costs (55–57). Specific personalized preventive interventions could be exclusively provided to high-risk individuals for developing a particular disease based on risk calculations. This could therefore lead to far more cost-effective outcomes (58), particularly through the avoidance of over-diagnosis and redundant treatment (59). Nevertheless, more cost-effective calculations are needed, especially for omics-testing technologies (55). Further research about behavioral science interventions will be needed in order to ensure a sustainable integration of personalized preventive interventions and behaviors (55).

The aim of the German National Cohort is to investigate the causes of diseases through long-term monitoring, to discover and validate new biomarkers, and to develop preventive measures. Progress in biomarker research will enable continuous improvement in the early detection of chronic diseases and their precursors (60).

## Implementational challenges of personalized prevention

The incorporation of personalized prevention into health practice remains limited. Healthcare systems face a variety of challenges that stand in the way of a successful realization of the full potential of this approach. One of the greatest long-term challenges is the implementation of policies. In their 2021 review, Trein and Wagner give an overview of the four most relevant policy challenges found in the literature for implementing precision health (including personalized prevention) in practice: (1) creating, maintaining and harmonizing an infrastructure for research, (2) building and fostering trust in precision health amongst citizens in general and patients in particular, (3) establishing regulatory frameworks to ensure cooperation and to avoid discrimination, and (4) integrating precision health into existing health systems (61).

### Genetic testing

In order to incorporate more genetic testing into medical procedures in the future, the acceptance of these technologies by the general public will be an important factor (62). Previous systematic reviews report mixed attitudes toward genetic testing, with a higher number of studies reporting positive attitudes (62, 63). Participants perceived benefits from genetic testing, especially for prevention and treatment of diseases (63). The primary areas of public concern relate to the privacy of genetic information, with a special fear of misuse in the form of genetic discrimination (GD) (62, 63). The most researched domains that could be affected by GD are insurance and employment settings (64, 65). One negative consequence of these concerns could be an unwillingness to undergo genetic testing among individuals high at risk, which could cause increased manifestations of disease and death (64). Numerous policies, laws and strategies have been developed in different countries to prevent GD in insurance and employment settings. These include GINA (Genetic Information Nondiscrimination Act) in the USA (64), and the GenDG (Genetic Diagnostics Act) in Germany (66). All of these “explicitly proscribe discrimination based on the DNA composition of an individual's genome” (64). However, the definition of genetic information used in these policies is greatly limited and does not include new precision health biomarkers like omics-technologies. Thus, current literature identifies an urgent need for developing international standards, collaborative initiatives, and revised national policies addressing GD in the future (64, 65). The fear of GD could lead to the use of DTC-GT approaches outside the regular healthcare system as a result of consumers hoping to bypass potential GD (66). The implementation of DTC-GT in healthcare may seem promising, but some concerns remain. These include worry that new technologies may not always be covered by anti-discrimination or data protection laws, and concerns of lacking medical communication of the results. Genetic counseling with genetic health professionals should always take place in order to adequately discuss genetic results.

### Education

For a successful implementation of personalized prevention in health care practice, a better understanding of personalized concepts and technologies among healthcare providers is crucial. The demand for appropriately trained health professionals is currently growing. Therefore, the continued development of educational programs and training in omics sciences, personalized communication skills and personalized prevention concepts in general will be of key importance. It will be both necessary to define core competencies that can be integrated into the curricula of health care professional training programs and to develop national policies and regulatory frameworks that ensure the integration of adequate personalized preventive practice (56).

Health literacy and an understanding of the benefits and limits of personalized preventive technologies like omics-testing is needed for citizen empowerment and engagement in both contributing to research and adopting new behaviors in everyday life (56, 57, 67). This will only be possible if educational, ethical, socioeconomic and cultural obstacles are correctly addressed and managed (55). These topics must be addressed cross-sectionally and therefore, perspectives from experts in different health care disciplines must be integrated (2, 55).

### Health disparities

Guaranteed access to healthcare for all is a crucial goal that will require focused and sustained effort. The elimination of health disparities will require system changes that promote health equities and access to high quality care for all (68). Social, ethnic, economic and regional disparities are still not appropriately addressed (67).

A lack of ethnic diversity in the data creates a disturbing potential for the exacerbation of already existing health disparities. For future research, it is therefore essential that study samples include diverse patient populations (different ethnicities and socially disadvantaged populations) to ensure that scientific advances benefit all populations (55, 68, 69). Besides biomarkers, data regarding environmental, socioeconomic, sociocultural, and behavioral factors should be collected for a holistic understanding of influences on the development of diseases (68). Furthermore, personalized telemedical approaches have the potential to reach populations that are otherwise difficult to reach and make it possible to collect more diverse digital biomarkers (59).

Few reviews have summarized strategies and recommendations for the implementation of personalized medicine into preventative care. The recommendations most mentioned in the literature include: (1) develop education programs for personalized prevention and the building of community awareness, (2) empower and engage patients, (3) develop infrastructure and information management systems, (4) value recognition for precision health, and (5) ensure access to care (67, 70). Table 3 presents different challenges and barriers for a successful implementation of personalized prevention in healthcare practice, along with potential strategies to overcome them.

**Table 3:** Challenges and barriers for a successful implementation of personalized prevention in healthcare practice and strategies to overcome those.

	**Challenges and barriers**	**Strategies to overcome barriers**
	Lack of education and awareness in healthcare providers as well as patients	- Professional education and training in personalized preventive concepts, quality standards for laboratories/ omics and genetic testing and public awareness are essential factors for the successful implementation of personalized prevention in practice (2)
	Health disparities in access to care; lacking diversity in data “Racial, ethnic, economic and regional disparities are not appropriately addressed” (67)	- Implement system changes in healthcare coverage to ensure equitable access to quality screening and treatment (68)
		- Include diverse populations in research (ethnic minorities and socially disadvantaged populations) to ensure that scientific advances benefit all populations by using innovations in personalized prevention to reduce and ultimately eliminate health disparities (55)
Implementation challenges		- Support research that focuses—in addition to biological underpinnings—on the complex interaction of behavioral and social determinants of health and health disparities (69)
	Management of big data, data safety and prevention of genetic discrimination Health system infrastructure and information management are not yet well equipped for handling the massive amounts and different kinds of information associated with personalized prevention (67)	- Develop and implement health policies that ensure data safety and the prevention of genetic discrimination
		- Patient confidentiality and privacy should always be a priority and at the forefront of healthcare innovation (59)
		- Development of infrastructure and information management systems
	Unclear areas of responsibilities; lack of time for primary-care givers	- Roles and responsibilities (e.g., risk assessment, genetic counseling, genetic testing) should be redistributed among the various health professions to improve work performance and standard of care (2)
		- Clarification and validation of the obligations and responsibilities of the research community, research participants, and the public
		- Collaboration and dissemination of high-quality ethical, policy and legal analyses (2)
Methodological challenges	Heterogeneity of study design lacking data addressing efficacy and (cost-) effectiveness	- Large prospective population-based studies with a large amount of data collected using standardized methods, together with biological samples stored in a biobank
		- Large studies examining the effectiveness of risk communication based on a broad range of newly discovered biomarkers (as opposed to risk communication based solely on traditional risk factors) in changing behavior in healthy individuals at increased risk (2)

### Implementation and resistance

Current prevention strategies and recommendations are largely based on the results from large population-based studies and target shifts in the entire population. Public health prevention is often viewed as an altruistic principle which values the benefit of the population above that of the individual (e.g., vaccination). Such population-strategies of prevention are in contrast with a personalized prevention approach. Thus, the implementation of personalized prevention strategies dependent at least in part upon the public health infrastructure (71).

## Future perspectives

Personalized prevention demonstrates great potential to increase the effectiveness of preventive interventions. However, achieving the successful integration of personalized prevention into everyday health care will require changes in health care systems. The pursuit of this goal will require all stakeholders to work to enable the implementation of personalized prevention. Along with improving the training of health practitioners, policies, regulations, and companies will also have to adapt. Some of the required changes will require long term implementation. These include structural changes in the healthcare system and the development of policies and legal foundations to address issues like health disparities and genetic discrimination. In terms of changes that may be achieved in the short term, we see great potential in advancing the education of health professions in omics sciences and enhancing citizen awareness and engagement. For the clinical setting, we recommend further development of personalized prevention techniques using predictive biomarkers as well as telemedical approaches in primary care institutions. Personalized prevention offers a variety of promising opportunities. Therefore, we encourage physicians, health policy makers, and public health professionals to consider and apply the key elements of personalized prevention into practice.

## Author contributions

KM obtained funding and critically reviewed the manuscript. SJ, CN, and KM conceptualized the review, contributed to manuscript revision, and have responsibility for final content. SJ and CN wrote the manuscript and did the literature research, study selection, and interpretation. SJ created the figures. All authors read and approved the final manuscript.
